# Lived and Self-Reported Experiences of Immersive Virtual Nature Environments Among Patients and Spouses in Palliative Home Care: An Exploratory Mixed-Methods Feasibility Study

**DOI:** 10.3390/healthcare14213637

**Published:** 2026-10-27

**Authors:** Catharina Norberg, Lisbeth Slunga Järvholm, Kristina Lämås

**Affiliations:** 1 Department of Nursing, Umeå University, S-901 87 Umeå, Sweden; kristina.lamas@umu.se; 2 Department of Epidemiology and Global Health, Umeå University, S-901 87 Umeå, Sweden; lisbeth.slunga-jarvholm@umu.se

**Keywords:** palliative care, home care, nature assisted therapy, nature environment, immersive virtual reality, attention restoration theory, symptom reduction, emotional well-being

## Abstract

**Highlights:**

**What are the main findings?**

• IVR-N was reported to provide a shift of focus away from emotional and bodily suffering in palliative home care.
• IVR-N was experienced as a valuable substitute for real-life nature experiences when restricted by life circumstances.

**What are the implications of the main findings?**

• IVR-N may provide distraction and experienced relief from pain, anxiety, and distress in palliative home care.
• Positive experiences of IVR-N may be influenced by how the virtual natural environment captures attention and evokes familiarity and memories.

**Abstract:**

Objectives: To explore lived and self-reported experiences of using immersive virtual reality with natural environments (IVR-N) among patients and their spouses in a palliative home care context. Methods: An exploratory mixed-methods feasibility design with purposive sampling was used. Data were collected through self-reported questionnaires, semi-structured interviews, and registrations from the VR headsets. Patients rated their symptoms and spouses rated their mental state, before and after using IVR-N. Descriptive analyses were used for quantitative data. Interviews were analyzed using qualitative content analysis. Quantitative and qualitative data were analyzed separately and subsequently integrated to provide a comprehensive understanding of the study findings. Results: The sample consisted of seven patients in palliative home care and four spouses, with a mean age of 76 years in both groups. Patients preferred natural environments that contained elements of activity or movement. There was a tendency towards lower symptom ratings in patients and more favorable ratings of mental state in spouses after IVR-N. Analysis of the interviews identified three themes: being in a place where tranquility becomes possible, connecting to nature via past experiences, and being prevented from engaging fully with nature. No adverse effects were reported by the participants. Conclusions: IVR-N may contribute to relieved symptoms in patients receiving palliative home care and improve mental state in spouses. The participants appreciated the feeling of being in another world and finding calmness, but they also preferred virtual environments that included some activity, to provide them with distraction. Larger randomized studies are warranted to confirm the results from this feasibility study in palliative home care.

## 1. Introduction

Individuals living with severe illnesses and limited life expectancy frequently experience a range of distressing symptoms resulting from both the disease and its treatment, including pain, nausea, anxiety, depression, and profound fatigue. The primary objective of palliative care is to alleviate these symptoms and improve quality of life. The concept of total pain underscores the multidimensional nature of suffering, encompassing physical, psychological, social, and existential dimensions [1]. A holistic approach addressing all dimensions is therefore essential in palliative care to ensure comprehensive symptom relief.

Natural environments are widely recognized for their positive effects on health and well-being [2]. For example, a study from northern Sweden demonstrated that forest exposure positively improved mental state in individuals diagnosed with exhaustion disorder [3]. Theoretical frameworks for the health-related benefits of nature assume that humans have evolved in natural environments and therefore have an inherited biological and psychological relationship with natural environments [4].

Two conceptual frameworks are often used to explain the beneficial effects of nature: attention restoration theory [5] and stress reduction theory [6]. Attention restoration theory, which was developed by Kaplan and Kaplan [5], focuses on cognitive recovery. The theory suggests that prolonged use of directed attention can lead to attention fatigue. Natural environments promote recovery through soft fascination, a form of effortless attention that supports reflection and attention restoration. Restorative environments are characterized by fascination, a feeling of getting away from it all, a sense of extent that promotes connectedness, and compatibility with individual needs and preferences [7]. Stress reduction theory, which was proposed by Ulrich et al. [8], focuses on nature’s affective impact. It posits that exposure to natural settings reduces stress via decreased sympathetic and increased parasympathetic activity. This stress relief, in turn, supports cognitive functioning [9].

For patients with advanced illness, access to natural environments is often limited or absent. Immersive virtual reality (IVR) offers promising alternatives by enabling simulated nature experiences. The effects of IVR in palliative care have been explored in quantitative studies and synthesized in systematic reviews. Mo et al. [10] concluded that IVR is both feasible and well tolerated in clinical settings; however, the strength of the evidence was constrained by the low methodological quality. In a more recent review, Gaina et al. [11] identified IVR as a promising intervention for alleviating discomfort among patients in palliative care, with the potential to reduce anxiety, stress, and pain to a degree comparable to or exceeding conventional treatments. The authors also suggested that IVR can be effectively integrated as a complementary approach within palliative care. However, the substantial heterogeneity in design, patient populations, IVR content, and outcome measures complicated direct comparisons and weakened the overall evidence base.

IVR in a palliative context has also been studied qualitatively. In a meta-synthesis of qualitative studies, Huang et al. [12] found that IVR enabled patients in palliative care to virtually access otherwise unattainable places and experiences, promoting relaxation, calm, and positive emotions. It also offered a means of escape from constrained circumstances and elicited meaningful life memories. Although minor side effects such as nausea, dizziness, and claustrophobia were reported, the overall findings support IVR as a potentially safe and effective intervention for symptom management and enhanced well-being in palliative care settings. However, the content of IVR sessions varied considerably, ranging from passive exposure to nature’s imagery to more interactive formats [12]. Notably, no systematic reviews or meta-synthesis have, as far as we have found, focused exclusively on IVR involving natural environments.

The ability to choose an environment that aligns with personal preferences may be important. Niki et al. [13] found that visiting memorable places through IVR significantly improved both physical and psychological well-being in several aspects. In contrast, participants who chose to visit places that they were interested in but had never seen before did not report similar improvements. Lloyd and Haraldsdottir [14] and Gerlach et al. [15] have also described the value of spending time in IVR environments that evoke precious memories.

The impact of severe illness extends beyond the patient and often places substantial emotional and physical strain on close relatives. Continuous support and care in home settings can lead to isolation, reduced social interaction and recreation, and diminished quality of life among caregivers [16]. Moreover, informal caregivers frequently report a lack of adequate support. IVR may offer opportunities for caregivers to rest and recover through recreational experiences. Quantitative findings suggest promising effects. Ordu and Yilmaz [17] reported that repeated IVR sessions featuring nature scenes or a virtual museum led to improved psychological well-being and a reduced caregiver burden [17]. Moreno et al. [18] found that a single IVR session lasting 7–8 min significantly reduced anxiety among caregivers in an end-of life care unit. Similarly, Letho et al. [19] observed improved well-being and quality of life following 10 min nature-based IVR (IVR-N) sessions administered over five days among informal caregivers in home-based palliative care.

A qualitative study [20] further suggests that IVR-N is feasible and acceptable for informal caregivers. Exposure to nature via IVR has been described as relaxing and calming, fostering mindfulness, and a sense of escapism [20].

Evidence for the use of IVR-N among patients and/or informal caregivers in a palliative care context remains limited. Although research in the field has increased rapidly in recent years, few studies focus on the known benefits of using familiar natural environments. Some studies focus exclusively on natural environments, but most employ mixed content such as the natural environment and public places [17,18,21], or integrate additional elements including interactivity [22], music therapy [23], breathing and self-compassion exercises [24], or guided imagery or relaxation in a natural environment [25]. To further understand the feasibility and perceived benefits of IVR-N, the present study focuses specifically on exposure to natural environments with ambient nature sounds. Given that familiarity may mediate positive experiences, the environments were selected from nearby geographical areas. In addition, to account for individual preferences, multiple types of natural environments were included.

The overall aim of this study was to explore lived and self-reported experiences of using IVR-N among patients and their close relatives in a palliative home care context, as well as to gain insight into the feasibility and safety of using IVR-N in such care settings and in larger future studies.

## 2. Methods

### 2.1. Design

This study is part of the Interreg Aurora research program “NATUREACH—Nature Reachable for All” (NYPS 20358051), which has the overall goal of developing and evaluating IVR-N for different client groups and health care professionals in the northern parts of Sweden and Finland. Participants in this study comprised a small group of patients, who were admitted to palliative home care, and their close relatives. The study should be considered as a feasibility study with a mixed-methods design. Quantitative and qualitative data were collected and analyzed separately. The findings were subsequently integrated to provide a more comprehensive understanding of the study findings.

### 2.2. Setting

The study was conducted in northern Sweden from April 2024 to March 2025. At the time of inclusion, all patients (*n* = 7) were receiving palliative care in their homes from an advanced home care team. One patient moved to a hospice during the study period because of a worsening condition. Six patients lived with a close relative; four of them agreed to participate in the study.

### 2.3. Recruitment

Two registered nurses with specialist training in palliative care at the palliative home care unit, both of whom were part of the study team, identified suitable patients for the study. Inclusion criteria for patients were aged 18 years or older, enrolled in a palliative home care unit, and having the ability to understand the information they were given. The exclusion criteria were: having a brain tumor, being at risk of seizure, experiencing vertigo symptoms, and having cognitive impairment. The clinical criteria were assessed by the two specialist nurses. They also had the opportunity to consult physicians at the care unit. Inclusion criteria for relatives were: being closely related to the patient and being involved in the daily care. The invited close relatives were all spouses and in the following text we use “spouses” when referring to the close relatives that participated.

Patient selection for the study was purposive; the specialist nurses contacted and offered participation only to those patients they deemed suitable for inclusion. The patients needed to be in a sufficiently stable condition and have a life expectancy that was not overly short. In total, ten patients were invited and seven of them agreed to participate.

### 2.4. Intervention

To ensure an appropriate and safe intervention using suitable approaches for the participants, an early dialog was initiated between the staff at the palliative care unit and the researchers. Two specialist nurses and two physicians from the care unit were included in the project team and participated from the planning phase onwards.

VR headsets (Pico 4 Enterprise, Pico Interactive, Beijing, China, Product Specifications | PICO4—PICO Global) were used during the IVR-N sessions. PICO 4 is a standalone VR headset equipped with dual LCD displays providing a resolution of 2160 × 2160 pixels per eye. The device incorporates pancake lens technology and offers a field of view of approximately 105°. It is powered by the Qualcomm Snapdragon XR2 platform and supports refresh rates of up to 90 Hz. The headset includes integrated spatial audio. All content was stored locally on the headset, allowing the intervention to be delivered without an internet connection. Participants could easily exit the session by directing their gaze toward a designated exit button in the virtual environment [26]. The patient and any spouse each had their own personal headset. The headsets were provided by the Interreg Aurora Natureach project, which conducted a joint procurement following a thorough market analysis to ensure good image quality, sound, and usability.

After informed consent and start of the study, the participants were individually introduced to their first IVR-N session by one of the specialist nurses. Participants were instructed to choose a comfortable position, either sitting in an armchair or lying in a slightly reclined position in bed. Participants could choose between ten different natural environments and were given time during the first session to familiarize themselves with them. Subsequently, two additional IVR-N sessions were conducted on separate occasions. During these sessions, the specialist nurse remained present to provide assistance with the VR headsets if required. Participants chose freely among the different nature environments, and each IVR-N session lasted about 15 min. If a spouse also participated in the study, the sessions were conducted simultaneously for the couple, but each participant independently chose their preferred environment. Typically, these three IVR-N sessions were conducted over the course of 1–2 weeks. After the third session, the participants were offered the opportunity to borrow the VR devices for independent use for an additional two weeks. Four out of seven patients and three of the four spouses did so. During this additional period, they generally used IVR-N once to three times a week.

All the nature environments were recorded in northern Sweden and Finland, with the intention that they would be familiar to most of the participants. The locations were carefully selected to represent a diversity of natural landscapes (forest, lake, sea, river, mire, meadow), with variations in season, weather conditions, and time of day [26]. This diversity was intended to accommodate individual preferences while optimizing the recovery potential in accordance with previous research [27].

The scenes were recorded with 360° cameras placed in a calm, scenic location that provided an open view, natural protection from behind, and an absence of distracting elements such as recording equipment, sound, objects and people that are not a natural part of the environment and that would disturb the nature experience. This setup was expected to simulate the feeling of sitting comfortably in a tranquil natural setting. Site-specific sounds such as wind, flowing water, and birdsong were recorded at the same time. Some of the environments also contained animals.

The specialist nurse was present during the first three sessions, observing the patient and noting any reactions. After the session, questions were asked about the experience and whether there had been any discomfort or adverse events.

### 2.5. Data Collection

Data were collected via semi-structured interviews, self-reported surveys, self-assessment of symptoms and mental state, and registrations made automatically by the VR headset during the sessions (e.g., type of natural environment that was used and time points).

Baseline data characterizing the participants were collected at the start of the study. Subsequently, quantitative data were collected before and after the second and third IVR-N sessions, respectively. Qualitative data, in the form of interviews, were collected after each participant had completed the entire intervention and returned the headsets. During all data collection, a specialist nurse, who also was a research team member, was present to guide and assist the participant as needed.

### 2.6. Quantitative Data

#### 2.6.1. Patients

At study inclusion, demographic and clinical data were collected and the Integrated Palliative Care Outcome Scale for patients (IPOS) was administered to describe the current clinical condition, symptoms and individual needs at baseline [28]. It was not used as an outcome measure in the study, but solely to describe the clinical situation at baseline. The translated Swedish version is described and validated in a study by Beck et al. [29]. IPOS is based on a three-factor structure to grade physical symptoms (10 items), emotional symptoms (4 items), and communication and practical issues (3 items). Each item is scored on a Likert scale of 0–4, with 0 indicating no problems and 4 indicating the worst possible situation. Participants were asked to assess their situation over the previous three days. The questionnaire is used for all patients as part of the unit’s clinical routines for palliative care. The form is usually completed in conjunction with an interview and a review of the questions together with healthcare staff, which was also the procedure in the study.

Before and after the IVR-N sessions, the patients rated four symptoms that are common in patients receiving palliative care: pain, anxiety, nausea, and breathlessness. These symptoms were rated on a numeric rating scale (NRS) from 0 to 10, with 0 representing no discomfort and 10 representing severe discomfort.

#### 2.6.2. Spouses

Among the spouses, mental state was assessed before and after the IVR-N sessions [3]. Six items were used; tenseness (tense—relaxed), fatigue (exhausted—alert), mood (sad—happy), irritability (irritated—harmonious), restlessness (restless—peaceful), and mental clarity (mentally distracted—clear-headed). A 10-point Likert scale was applied, with a high score indicating a favorable condition.

The instrument was originally developed during a study involving mentally exhausted individuals participating in a forest-based rehabilitation study. The instrument was developed with the aim of being user-friendly, consisting of a few questions, and being quick to complete. Its design and content were inspired by similar validated instruments, such as POMS and ZIPERS, but also based on the documented psychological effects of spending time in natural environments [7,9]. The instrument has not yet undergone a rigorous validation study. However, we considered the instrument to be suitable as a quantitative outcome measure for spouses providing home-based palliative care to their partners, particularly for assessing changes before and after an IVR-N session.

### 2.7. Qualitative Data

After all IRV-N sessions and collections of the headsets, participants who had agreed to be contacted by telephone were invited to take part in individual semi-structured interviews (Supplementary File S4). These interviews were conducted by the first author (CN), who has an in-depth understanding of palliative care, although without being involved in the participating patients’ care. Patient interviews took place in the participants’ homes (n = 3), via the telephone (n = 1), or at a hospital ward (n = 1). Spouses who had used the IVR-N were interviewed individually in their homes (n = 4).

A semi-structured interview guide was developed and discussed with the study team. Topics covered the participants’ experiences during and after the VR sessions, including both emotional reactions and bodily experiences. Participants were asked about their preferences concerning the available natural environments and suggestions for improvements. Questions were also asked about the use of the VR headset. See Supplementary Interview Guide. The interviews were audio-recorded and lasted between 8 and 30 min, with a median of 29 min for patients and 20 min for spouses.

### 2.8. Analysis

#### 2.8.1. Quantitative Analysis

The quantitative data and analyses covered the second and third assisted IVR-N sessions in accordance with the study protocol. Each session was analyzed separately, as the order of the sessions could potentially influence the results. Due to the low number of participants recruited to the study, the quantitative data were only analyzed descriptively in terms of mean values and standard deviations. Each participant’s data is reported in the Supplementary Files S5 and S6. There was no missing data to handle.

#### 2.8.2. Qualitative Analysis

The qualitative data and analyses covered the entire study, including two weeks of independent use, which applied to four patients and three spouses.

All interviews were transcribed verbatim (CN) and read multiple times to ensure familiarity with the data. The analysis was conducted in accordance with the approach described by Graneheim and Lundman [30], involving the identification of meaning units, condensation of these units, formulation of codes, and the subsequent organization of codes into categories, subthemes, and themes. This process of coding and categorizing was carried out collaboratively (CN, KL), with ongoing discussions and critical reflection among the authors to enhance analytical rigor (CN, LSJ, KL). The analysis was iterative in nature, involving constant movement between meaning units, subthemes, and themes to ensure the credibility and trustworthiness of the findings (Supplementary File S7).

Qualitative software tools were not used, only an Excel 365 to sort and organize the qualitative data. The interviews were conducted in Swedish, and the quotations were translated into English collaboratively by the authors.

#### 2.8.3. Integrative Analyses

An integrative mixed-methods analysis was conducted, in which findings from the quantitative and qualitative data were systematically compared and combined. The qualitative findings were used to explain, deepen, and contextualize the quantitative results, thereby enabling a more comprehensive and nuanced interpretation of the data. Analysis with integration of quantitative and qualitative data commenced after the study had concluded.

### 2.9. Ethical Considerations

This study was conducted in compliance with the Declaration of Helsinki (https://www.wma.net/policies-post/wma-declaration-of-helsinki, accessed on 25 May 2026) and approved by the Swedish Ethical Review Authority (2024-00433-01). Written informed consent was obtained before participation by one of the specialist nurses. Participants received both written and oral information about the study and were informed that participation was voluntary and that they could withdraw from the study at any time without providing a reason and without any consequences for their care (Supplemetary Files S2 and S3).

Conducting research in palliative care presents challenges, and the ethical appropriateness of such research has been discussed. Patients receiving palliative care frequently experience fatigue and other distressing symptoms, while their relatives, who often act as informal caregivers, may be emotionally and physically exhausted. This raises the important ethical question of whether it is justifiable to invite these individuals to participate in research studies.

Some authors have argued that patients in palliative care are unlikely to benefit directly from the outcomes of research [31]. However, a literature review encompassing eight studies suggests that both patients and their relatives may derive personal benefits from participating in research, and that they often express a desire to contribute [32].

To safeguard participants’ autonomy and well-being, ethical principles and guidelines have been established to ensure that individuals can make informed and voluntary decisions regarding research participation. The staff in the palliative care unit assessed the patients as having sufficient cognitive capacity to comprehend the information provided and to give written informed consent to participate.

## 3. Results

The participants and their involvement in different parts of the study are described in Table 1. Participant recruitment was conducted by the responsible specialist nurse and was based on an assessment of the patients’ clinical condition and suitability for participation. Ten patients were deemed potentially suitable and were asked if they wished to participate. Seven patients and four of their respective spouses agreed to participate. There were no dropouts or data losses during the collection of quantitative data, which was carried out at the second and third assisted IVR-N sessions.

Subsequently, the participants were offered the opportunity to borrow the equipment for an additional two weeks; four patients and three of their partners accepted this. According to headset log data, they used the IVR-N approximately 1–3 times a week during this extended period. Finally, five patients and four of their partners participated in qualitative interviews. No adverse events were reported during the study. However, one participant reported an undesirable experience evoked by the natural environment.

### 3.1. Quantitative Results

Characteristics of the participants are given in Table 2. None of the patients or spouses had previous experiences using VR techniques.

Table 3 shows the natural environments chosen by patients and spouses, respectively, at the two IVR-N sessions when ratings of symptoms and mental state were performed. The patients often chose environments with animals and birds, and quite often also environments during wintertime. Spouses varied more in their choices of environments.

**Figure 1:**
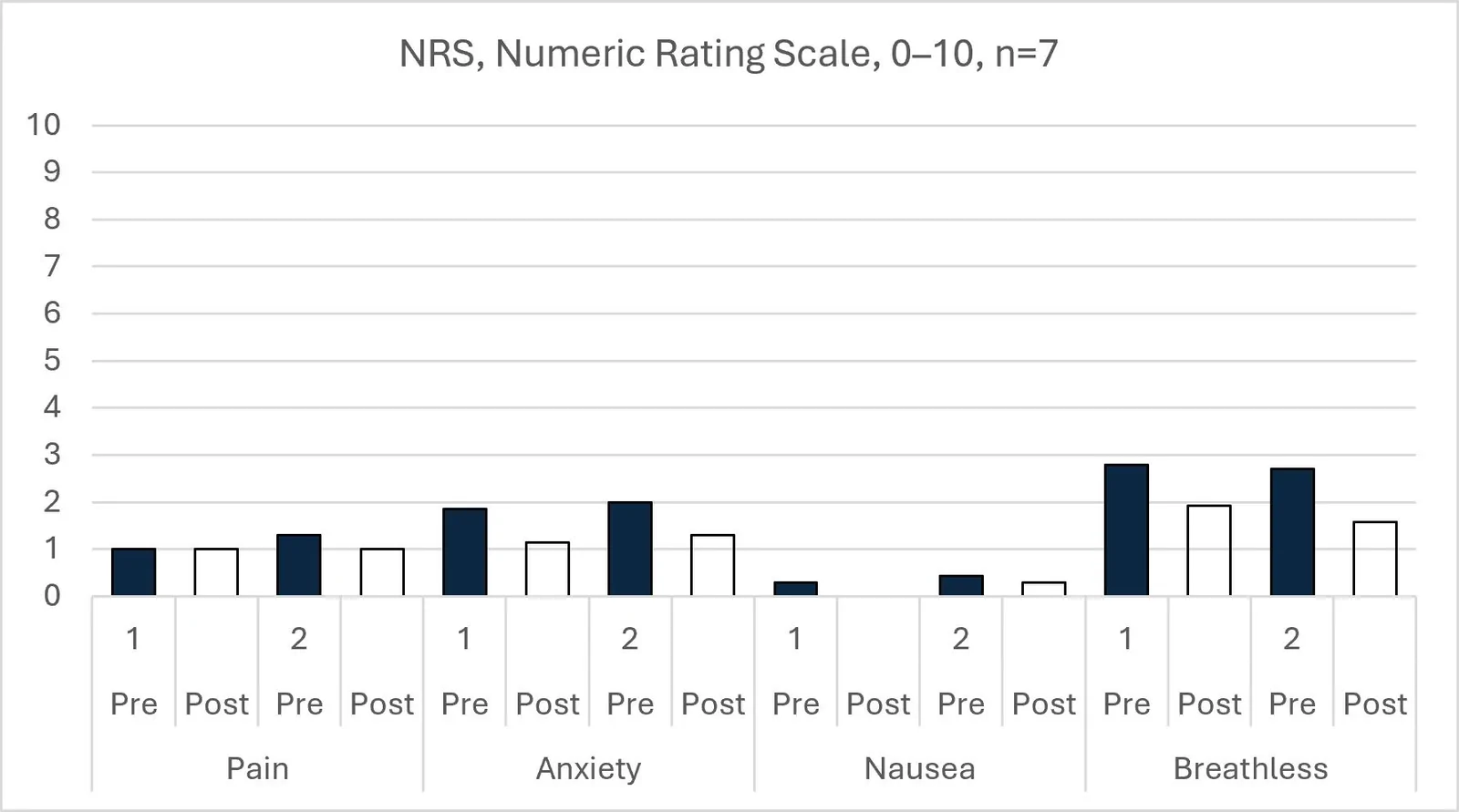
Mean symptom scores assessed with a numeric rating scale (NRS) in patients receiving palliative care before and after being exposed to immersive virtual reality with natural environments (IVR-N). All patients had two IVR-N sessions with data collection a few days apart. A high score indicates a high grade of symptoms.

Results of symptom ratings in patients are illustrated in Figure 1. The ratings were generally low before the IVR-N sessions and tended to be even lower after the sessions. Each participant’s ratings and the group’s numerical mean values are presented in Supplementary File S5.

**Figure 2:**
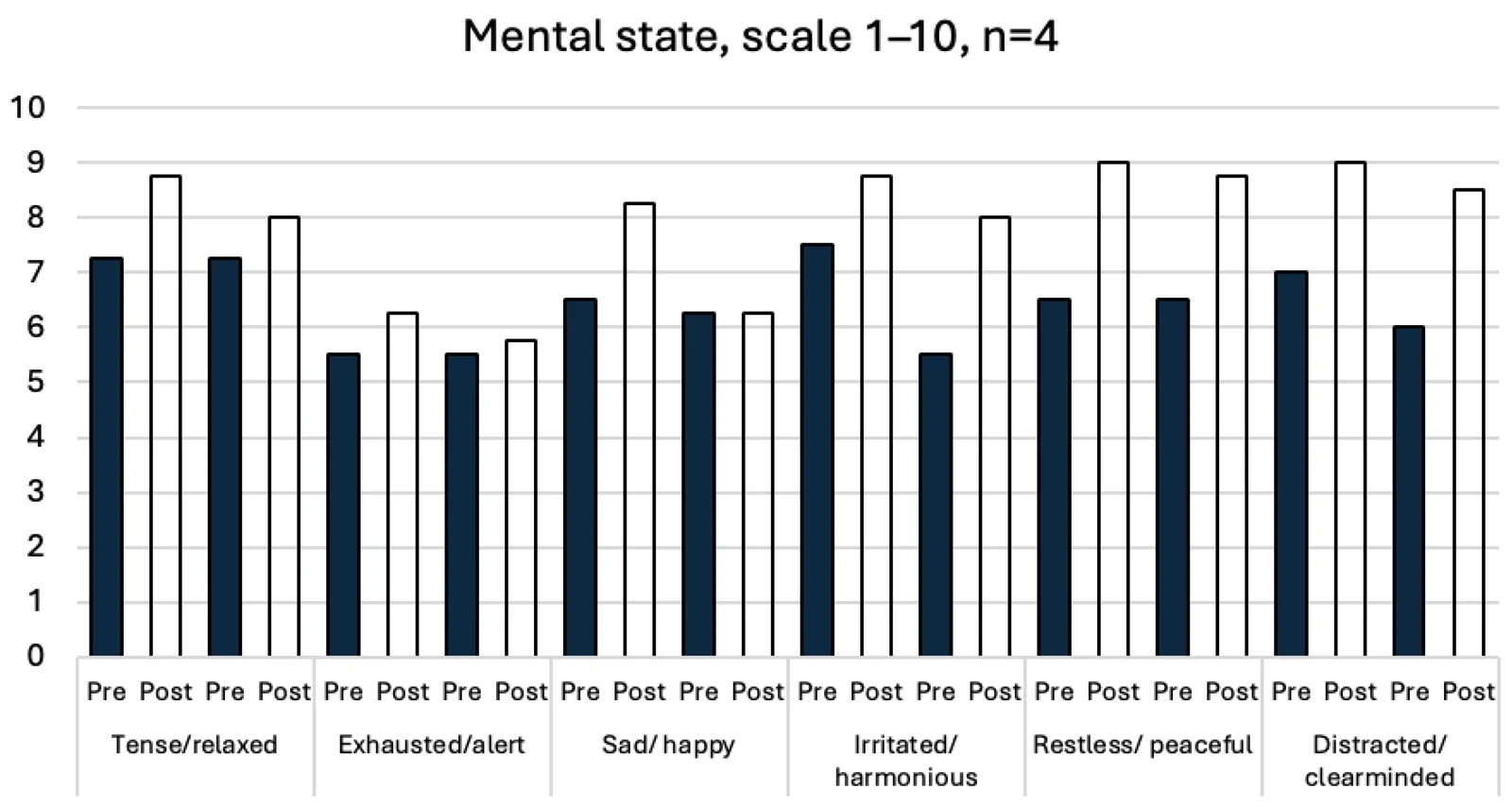
Mean mental state scores in spouses before and after being exposed to immersive virtual reality with natural environments (IVR-N). All participants had two IVR-N sessions with data collection a few days apart. A high score indicates a favorable mental state.

Results from ratings of mental state in spouses are illustrated in Figure 2. There were indications of higher and more favorable mental state ratings after the IVR-N sessions. Each participant’s ratings and the group’s numerical mean values are presented in Supplementary File S6.

### 3.2. Qualitative Results

Seven patients and four spouses completed the IVR-N intervention and the quantitative measurements. Five patients agreed to participate in the individual interviews, one patient did not agree, and one patient died before the interview. All four spouses participated in the interviews.

The analysis of the interviews revealed three themes describing the experience of IVR-N: *being in a place where tranquility becomes possible*, *connecting to nature* via *past experiences*, and *being prevented from engaging fully with nature* (Table 4) (Supplementary File S8).

#### 3.2.1. Being in a Place Where Tranquility Becomes Possible

Spending time in IVR-N was interpreted as *being in a place where tranquility becomes possible*. The participants described being in IVR-N as being in another world where they found peace and calmness. They expressed relief from emotional as well as bodily suffering when they spent time in this other world. This theme comprised four subthemes: *being in another world*, *finding peace and calmness*, *being released from emotional suffering*, and *being released from bodily suffering*.

##### Being in Another World

The participants reported being surprised by the sensation of entering “another world” when they used the headset. The IVR-N was perceived as realistic, and the ability to look around freely in all directions was described as remarkable. This immersive experience appeared to create a strong sense of being situated in nature, which was described as facilitating a temporary disengagement from everyday life and was quickly perceived as a new, alternative reality that was described as being in a “good place”. The alternative reality experience may have been supported by the design of the headset, which was experienced as comfortable and well tolerated, with no adverse effects such as dizziness or nausea, even among individuals with limited visual fields or tinnitus.

“Since it’s all around me, it felt like being there … I got into the same mood I’ve experienced out in the woods.”

(Patient 1)

“…by the sea I loved to curl up in a blanket and listen to the sea…I got a feeling of being there.”

(Spouse 2)

##### Finding Peace and Calmness

Spending time in IVR-N appeared to make it easier for the participants to unwind and relax. They described the natural environment as evoking a sense of peace and calmness which they found to be soothing and pleasant. The sounds of nature were highlighted as particularly important, especially in environments where there was a lack of visual activity, as the peaceful surroundings allowed the auditory elements to come to the forefront. Sounds such as waves breaking against the shore, birds chirping, owls hooting, and wind rustling through the trees were described as emotionally moving. These natural sounds seemed to help the participants stay focused on IVR-N and prevented their thoughts from drifting back to their difficult situation. For some natural sounds were described as having an even deeper impact than the visual aspects of the environment. Unfortunately, some described difficulties with adjusting the sound volume resulted in an auditory experience that was weak or, at times, nearly absent.

“I felt that the sound moved me more than the images did. The sound was so powerful—I didn’t expect to be so touched by it, compared to the images.”

(Spouse 4)

“It was hard [to hear the sounds]. And I pressed that plus button. I almost never heard any sound in any of the pictures. Maybe there was a little sound with the waves splashing. You could hear that a bit. But it wasn’t really anything I missed. But when you did hear some birds chirping once in a while by chance, then you reacted to the sound. Because there wasn’t any sound very often. So, when I heard a sound, I reacted to it. Like, oh wow, a sound.”

(Patient 3)

##### Being Released from Emotional Suffering

The participants described the strained situation they were living in. Being aware that their remaining time was limited was described as evoking dark feelings and led to brooding. It seemed that the natural environment served as a distraction; for a moment, they described being relieved from their suffering, which they valued greatly. The darkness and thoughts about the end of life seemed to briefly be dispelled, offering a temporary rest from their burdensome circumstances and feelings of sadness and worry.

“...having a break and thinking about something else is very important for me. It’s easy to get stuck in these rather dark thoughts. That’s quite hard. But that feeling disappeared after a little while when I was sitting there with those glasses on.”

(Patient 2)

“We could relax, we could sit there for about fifteen minutes... just look at those images and forget all the boring stuff that was behind us... we got a break, that’s what I felt.”

(Spouse 3)

##### Being Released from Bodily Suffering

The patients described how their body reminded them of their current illness and life situation; one symptom that was frequently mentioned was shortness of breath. IVR-N seemed to help them move their focus away from bodily suffering. They described the feeling of being present in IVR-N, noting that their body became more relaxed. Their shoulders relaxed, breathing became calmer, and the shortness of breath was less intrusive.

“… these last few days I’ve been experiencing terrible shortness of breath. It feels like I’m going to suffocate, like there’s no way to get air in or out. And then, being able to disconnect from that just by sitting and looking out over the sea, or something like that… that amazed me.”

(Patient 2)

“I felt relaxed and almost unaware of my body…it was soothing and calming to look at the images—especially when there was activity in the environment.”

(Patient 3)

#### 3.2.2. Connecting to Nature via Past Experiences

Being in IVR-N was described as a way of *connecting to nature* via *past experiences*, as it evoked memories that influenced the experience in several ways. While spending time in IVR-N, the participants described reliving earlier life events based on their memories evoked by the virtual environments. Encountering an environment that felt familiar also seemed to elicit a sense of at-homeness. However, this familiarity and the memories it triggered simultaneously seemed to remind them of the loss of opportunities associated with their current life situation. This theme comprised three subthemes: *reliving past experiences*, *feeling at home*, and *grieving lost opportunities*.

##### Reliving Past Experiences

Spending time in IVR-N was described as evoking memories from earlier in life. The participants said that they preferred environments that meant something to them, often scenes that held precious memories from childhood. They talked about the beach by the summer house, the cows in the meadow, and other familiar scenes. This sense of recognition seemed to be important. Recognizing the environment was described as bringing back emotions associated with earlier experiences and seemed to transition into the “other world” easier. However, reminiscence also meant that negative feelings could be triggered by memories of unpleasant experiences. For example, a frightening memory of walking on a mire and getting stuck in a muddy area was described as influencing how the mire environment was experienced in IVR-N. These emotions from earlier in life seemed to shape how the virtual landscapes were experienced, both positively and negatively. Through these memories, the participants described a feeling of truly being there again and reliving past experiences from vacations, travels, and everyday life.

During the sessions, the patients and their spouses used IVR-N, each with their own headset, allowing them to share memories with each other. This opportunity to jointly recall and relive shared memories and stories was described as valuable and meaningful. It made it possible to connect both with the natural environment and with each other.

“I especially liked looking at the cows… because it reminded me of my childhood. I mean really—that’s exactly what it looked like when I was a kid. And the cows used to roam free, so you could run into them and things like that.”

(Patient 2)

“It brings up those shared memories and shared stories—of course, that’s really great.”

(Spouse 1)

##### Feeling at Home

As well as describing the memories that were evoked, the participants also talked about how nature had been a place of inspiration, recreation, recovery, exercise, shared activities, and a kind of free zone where one could just exist without needing to consider anyone else. Even when no specific memories surfaced, the IVR-N seemed to evoke a sense of recognition and familiarity that the participants described as central. The forest, in particular, was spoken of as a setting that had given them a feeling of security and peace. The natural environments shown through the VR headsets were described as familiar to the participants and seemed to convey a sense of being in a safe place; a feeling of being at home.

“I heard the familiar rustling from the trees and … felt safe and very much at home.”

(Patient 2)

“I liked to look at the natural environments because they were familiar and reminded me of my childhood.”

(Spouse 3)

##### Grieving Lost Opportunities

Being reminded of earlier experiences through IVR-N seemed also to bring a touch of sadness. The scenes were described as recalling opportunities that no longer were possible in the current situation, with the end of life approaching and a body marked by illness that limited what could be done. When the reality of this loss became clear, it seemed to evoke a sense of grief over missed opportunities in life. At the same time, the participants also described feeling grateful for the experiences they had and seemed to find a sense of reconciliation with their life situation.

“… mainly I’m thinking about my illnesses… that it might not be possible to visit these environments in the future… Now it’s over, like everything else that ends when you get sick. I miss this environment, but it’s more that you feel that you’ve had it, you’ve had these experiences during your life, and it feels great that you’ve had it.”

(Patient 3)

#### 3.2.3. Being Prevented from Engaging Fully with Nature

The participants described how certain circumstances could hinder their ability to engage with the natural environments and achieve relaxation. A lack of attention-capturing elements in the natural environments was expressed to lead to the re-emergence of stressful thoughts, while technical shortcomings diminished realism and became distracting. This theme of *being prevented from engaging fully with nature* was described through two subthemes: *feeling a lack of ease in an overly calm environment* and *finding IVR-N to be a complement rather than a replacement*.

##### Feeling a Lack of Ease in an Overly Calm Environment

The participants expressed a desire for more activity within the IVR-N. They emphasized that some form of sensory engagement was necessary to create a sense of tranquility and relaxation. Elements in the environment that could capture their attention and spark curiosity—such as rippling water, waves against the shore, animals moving, or birds singing—were experienced as soothing. In contrast, environments that were too calm and lacked stimulation were described as making it difficult for the participants to detach from their stressful everyday lives and enter a relaxed state. An overly quiet setting could even lead to an undesirable experience: they reported becoming bored, their thoughts wandered, their worries increased, and feelings of anxiety arose. As a result, the momentary relief of forgetting their current situation did not occur.

“This type of calm nature environment was no help for me; it was more like the opposite.”

(Patient 7)

“I was still sitting and thinking about the same thing; I just couldn’t let go.”

(Spouse 4)

##### Finding IVR-N to Be a Complement Rather than a Replacement

None of the participants had any previous experience with IVR, and they expressed fascination at how lifelike the natural environments appeared in the VR headset. The participants described IVR-N as an enjoyable break, like taking “an excursion from their kitchen table”, and as something pleasant to do together with their partner. Although they experienced the natural environments as realistic and creating a feeling of “being in another world”, they repeatedly emphasized that IVR-N could never replace real nature experiences. For example, they pointed out that it was only possible to observe what the camera provided. Even with a 360° camera, animals sometimes moved out of view, and they could not follow them, which was described as frustrating.

Outdoor nature was described as having been an important part of the participants’ lives. While IVR-N was described as a valuable substitute when life circumstances limited access to the outdoors, it was considered secondary to real nature. The participants explained that when their stress levels were high, a walk in real nature, hearing birdsong and being physically present, could be relaxing and provide relief, whereas sitting down with IVR-N did not offer the same calming effect.

“... because I can’t walk that far now. So, I can use this [IVR-N] as a kind of help instead of going there myself, I could use these kinds of videos. It’s not enough, of course, but at least it’s something that helps.”

(Patient 1)

“So, all these little breaks and outings, even if they just happen at the kitchen table at home, they really mean a lot. I think it adds something different, something out of the ordinary. And for him… well, he’s always been used to being outdoors. We’ve always walked and walked and walked, and he’s worked in the forests … So of course it’s nice for him to see these things, but it can never truly replace actually getting outside.”

(Spouse 1)

### 3.3. Integrated Results

The quantitative results showed a slight tendency towards reduced symptoms of anxiety and breathlessness among the patients. The qualitative data complemented these results by offering a richer understanding of patients’ experiences, particularly regarding breathlessness. While spending time in IVR-N, the patients described a general bodily relaxation accompanied by slower breathing. The sensation of breathlessness was reduced, and the immersive natural setting appeared to temporarily distract them from experiencing their illness.

The quantitative results also indicated a general trend of improved mental state among the spouses after spending time in IVR-N, specifically reflected in increased feelings of relaxation, alertness, happiness, harmony, peacefulness, and clear-mindedness. The qualitative findings supported these results, showing that the participants experienced relief from emotional strain and a sense of calm and peace, but also provided a more nuanced perspective. It is not self-evident that IVR-N will universally provide a calming experience for patients receiving palliative home care and their spouses. Instead, environments that evoked a sense of familiarity were preferred, and some degree of sensory engagement appeared to be necessary. When such engagement was lacking, the sessions sometimes triggered anxiety, allowing thoughts about their difficult situation to continue circulating in their mind (Supplementary File S9).

## 4. Discussion

This study explored lived and self-reported experiences of IVR-N among palliative home care patients and their spouses, as well as the feasibility of using the method for this fragile patient group receiving care at home. The recruited study population was relatively small due to the fragility of the client group and the difficulty of recruiting individuals with sufficient strength. Despite the small sample, the results contribute important insights and experiences in an area where existing knowledge is scarce.

There were small indications of decreased physical symptoms in patients and improved mental state in spouses after using IVR-N. The patients were evidently well-managed from the outset, as they exhibited relatively low scores on the IPOS scale.

Engaging with IVR-N was described as entering a place where tranquility could be found, and like being in “another world” that evoked calmness and peace, relieved emotional and physical strain, and seemed to provide distraction from the difficult situation. The findings suggest that connectedness to nature through IVR-N was facilitated when the participants were presented with natural environments that felt familiar. Such environments were said to evoke cherished memories and a sense of homeliness. Although the participants expressed grief over being no longer able to experience outdoor nature, this was accompanied by feelings of gratitude for past experiences.

The participants also described some barriers to engaging with IVR-N. It was emphasized that overly serene environments with minimal sensory stimulation sometimes evoked anxiety, as the lack of distraction allowed worries about their situation to resurface. Additionally, some limitations related to technology seem to impede full engagement. Unlike real nature, where individuals can freely choose their position, location, and focus, IVR-N limits attention to what is captured by the stationary camera. Thus, the participants noted that although IVR-N may serve as a valuable complement, it can never fully replace the experience of being in real nature. 

### 4.1. Physical Symptoms

Patients’ pre-IVR-N self-ratings of physical symptoms were low, which was probably due to well-functioning palliative treatment. A tendency towards even lower symptom scores after IVR-N was noted, especially for anxiety and shortness of breath in the quantitative finding. It was also described in the qualitative findings that participants expressed released from bodily suffering. Three of the patients had severe lung disorder as their main disease. These results are consistent with findings in previous systematic reviews which showed reduced symptoms after use of IVR in palliative care [10,11,33]. However, due to floor effects, this study faced obvious difficulties in demonstrating further symptom relief in the quantitative measurements.

#### 4.1.1. Feeling Calmness and Peace

A tendency toward increased positive emotions associated with spending time in nature through IVR was observed in both in the quantitative and qualitative findings. In particular, the participants emphasized the calming and peaceful qualities of the experience. This aligns with previous research, as systematic reviews have shown that personalized IVR interventions in palliative care settings can reduce anxiety and enhance overall well-being [11,12,33]. However, a central limitation of many systematic reviews on IVR is the considerable heterogeneity in environmental content. The studies by Gaina et al. [11], Huang et al. [12], and Martin et al. [33], for example, included a wide variety of virtual settings, such as natural environments, visits to locations selected from Google Earth, or even roller-coaster simulations. IVR is frequently highlighted as a promising therapeutic tool, yet relatively little attention is given to how the specific characteristics of virtual environments influence user outcomes.

#### 4.1.2. The Role of Reminiscence

Recognition and familiarity appeared to play an important role when the participants selected natural environments in IVR-N. The participants described choosing environments associated with cherished memories which elicited positive emotions. In line with previous research, despite the variability in IVR content, personalization consistently emerged as a key factor influencing the effectiveness of IVR interventions [27].

One possible explanation for improvement in psychological symptoms could be the concept of reminiscence [33], which has been shown to decrease anxiety and depression and to improve quality of life among cancer survivors [34]. Reminiscence emerged as a salient component in the present study. The importance of prior experiences in enhancing the effect of a natural environment has also been highlighted by Basu et al. [35]. Drawing on attention restoration theory [7], Basu et al. [35] argue that restorative qualities depend on the capacity to experience fascination. Fascination is shaped through an interplay between the environment, prior experiences, and an individual’s current state. Thus, experiencing recognition and familiarity may be important for receiving a positive experience from IVR-N.

#### 4.1.3. Experiencing At-Homeness

According to the participants, the sense of recognition was so vivid that it evoked feelings of “feeling at home” when engaging with certain natural environments in IVR-N. The existential experience of feeling at home, or “at-homeness,” is a metaphorical concept that has been extensively discussed in the Scandinavian nursing literature and is considered an aspect of existential well-being [36]. In life-threatening situations, this existential sense of at-homeness is often threatened (e.g., [37]). The concept of at-homeness appears to have multiple connections to our findings. At-homeness comprises three dimensions: (a) being safe through reduced experience of illness and altered time perception, (b) being connected through reunifying ways of relating, and (c) being centered through recognition of oneself [36].

The dimension of being safe resonates strongly with our results. Participants described moments of release from emotional and physical suffering, as well as a sense of transcending time and space. These experiences are what Öhlén et al. [36] identified as contributing to a feeling of safety and thereby fostering at-homeness. The dimension of being connected through reunifying ways of relating refers to connectedness with oneself, others, and one’s environment. A clear sense of connectedness to the natural environment was evident in our findings. The natural scenes also elicited memories that may be interpreted as reconnections with earlier versions of oneself and with significant others, evoking a sense of belonging and reunion (cf. [36]). Finally, being centered through recognition of oneself may also be interpreted in relation to the memories evoked during the IVR-N sessions. These memories appeared to offer the participants an opportunity to recognize themselves as continuous beings; that is, individuals who remain the same despite illness and decline. This aligns with descriptions in the nursing literature of how recognition of one’s past self can support existential well-being (cf. [36]). It is therefore reasonable to believe that spending time among familiar scenes in IVR-N may contribute to existential well-being in the palliative phase.

#### 4.1.4. The Role of Distraction

The findings indicated barriers to experiencing a meaningful connection with nature through IVR-N. According to the participants, limited sensory stimulation sometimes allowed thoughts about their difficult circumstances to re-emerge, occasionally eliciting anxiety. Within attention restoration theory, soft fascination—defined as effortless, involuntary attention—is central to the restorative effects of natural environments, as it reduces cognitive load while enabling reflection [7,35]. However, for individuals facing life-threatening illness, who may already engage in heightened introspection, additional opportunities for reflection may be less beneficial than distraction or temporary psychological relief from the emotional and existential strain. In line with this, distraction has been identified as a key mechanism underlying the effects of IVR in palliative care [38].

Previous studies indicate variation in users’ experience of IVR; environments that evoked personally meaningful or cherished memories were described as calming and beneficial, whereas non-preferred environments were experienced as boring [15,39]. However, Norberg et al. [40] reported that participants taking part in a multimodal lifestyle program described being bored by insufficient activity within IVR-N, possibly reflecting expectations shaped by more dynamic virtual experiences such as nature films. On this basis, the authors emphasized the importance of managing expectations by clearly informing participants about what to expect from the IVR-N and allowing time for adaptation to a serene virtual environment.

The present findings show that a serene environment sometimes seemed not to capture the participants’ interests and thereby did not consistently function as a source of distraction, and in some cases even elicited anxiety instead. Notably, these experiences were not clearly reflected in the quantitative data, suggesting that reported experiences may represent transient concerns rather than sustained distress. Nonetheless, this highlights the importance of tailoring IVR content to individual needs and psychological states. While familiarity appears central, the optimal level of sensory engagement and activity likely requires careful individual calibration to promote relaxation and well-being.

#### 4.1.5. IVR-N a Substitute for Real Nature 

The present findings suggest that IVR-N may serve as a meaningful substitute for individuals who are unable to access real nature, even though it cannot completely replace real-world nature experiences. A review of nature-based VR as a method of health promotion [41] specifically highlighted the challenge of developing realistic natural environments using a VR technique. Some participants in our study reported that they could not hear nature sounds because of hearing impairment or technical shortcomings. In a study investigating the effect of VR nature with and without nature sounds, the participants perceived the silent virtual forest as unnatural [42], indicating that combining multiple senses may be important for creating a realistic nature experience.

#### 4.1.6. Methodological Considerations

This study was conducted within the context of palliative home care, a setting that remains relatively underexplored in previous IVR interventions. Research in home care for people with advanced or life-threatening illness presents inherent challenges, primarily due to limited duration and frequency of healthcare professionals’ visits. In the present study, the interventions were administered by registered nurses with specialist training in palliative care, which may complicate implementation given the constraints on visit time and frequency. However, the headset is relatively easy to use and could likely be offered without ongoing, long-term assistance from palliative home care staff.

The IVR-N in this study only included environment recordings that had been produced in nearby regions in northern Sweden or Finland, providing participants with the opportunity to recognize the natural environments [26], and to allow them to recall experiences from previous real nature visits. Exposure to carefully selected natural environments based on previous knowledge of health-promoting experiences is, to our knowledge, unique in studies of IVR in palliative care. Adaptations according to the individual’s previous experiences and current needs is suggested to be important when using IVR as a complementary treatment.

Another important part of this study was that a close relative living with the patient was also invited to participate in the study and use IVR-N for their own recovery and increased well-being. Each participant had access to their own IVR headset, which meant that a couple could simultaneously participate in the same IVR-N environment and share their experiences and memories (Supplement File S1).

### 4.2. Limitations

Eligible participants in this study were identified and invited to participate by a registered nurse with specialist training in palliative care. Assessment of the inclusion criteria was based on clinical expertise. Nevertheless, the possibility of gatekeeping cannot be completely ruled out and may have introduced selection bias into the study.

This study had a limited sample size due to the vulnerability of the target population. Patients invited to participate in the study needed to be relatively clinically stable. This may affect the generalizability of the study. The participants’ characteristics are described in as much detail as possible, to enable readers to assess the credibility of the study and to evaluate the applicability of the findings to other contexts. As participation was voluntary, a potential novelty effect associated with the use of IVR-N cannot be entirely excluded and may have influenced the transferability of the findings. The quantitative results are presented solely in a descriptive manner and provide an indication that should be interpreted with caution and in conjunction with the qualitative data. In addition, the feasibility design did not include a control group or long-term follow-up. Consequently, no conclusions can be drawn regarding the durability of the observed positive experiences, although it is reasonable to believe that the intervention should have provided only temporary relief.

The vulnerability of the participants and the advancing nature of their illness were factors that prevented two persons from taking part in the interview. It is difficult to draw conclusions about the effect from the missing interviews. However, we found that the qualitative data were rich in both content and variation and provided a deepened understanding of the participants’ self-rated experiences.

Further potential limitations could be interview-mode differences, social-desirability bias, and researcher allegiance, all of which may have influenced participants’ responses and the interpretation of findings.

To enhance reflexivity and minimize potential bias, analytical decisions were discussed among the research team, interpretations were continuously compared with the original transcripts, and consensus was reached through iterative discussions. Quotations are provided to allow readers to evaluate the credibility of the interpretations [30] (Supplementary File S10).

## 5. Conclusions

The findings from this study indicate that nature-based experiences delivered through VR can recreate the sensation of being outdoors, thereby offering individuals a valuable source of respite from physical limitations, environmental constraints, and mental stressors in palliative home care. Even though direct contact with natural environments is generally preferred, IVR-N may serve as an alternative when such contact is not feasible, while not fully replacing experiences of real nature. There was a slight tendency towards less anxiety and breathlessness among patients receiving palliative home care and improved mental state among spouses.

Larger randomized controlled studies are needed to further evaluate the potential effects. Consideration should be given to the content of the IVR-N and the specific needs of each patient. The use of familiar natural environments can evoke memories of past experiences and provide shared experiences between patients and spouses.

### Implications for Clinical Practice

Although the participants had no previous experience with VR technology, they found the equipment easy to use and did not report any adverse effects during or after the intervention. The results suggest that IVR-N can be a useful supportive intervention and provide some relief from experienced symptoms in palliative care. However, certain conditions should be aimed for; user experiences seem to be enhanced if natural environments evoke a sense of familiarity and memories and include elements that are sufficiently stimulating to capture and sustain attention. IVR-N could potentially be used as a distraction intervention and may help reduce patients’ experiences of pain and anxiety in connection with treatments and medical procedures [41]. We also propose introducing IVR-N at a relatively early stage of the disease, so that handling the equipment and learning the method do not become overly demanding.

## Figures and Tables

**Table 1:** Description of recruited participants, dyads of patients and spouses, and involvement in the different parts of the study.

Participants	First Part of Study with Three Assisted IVR-N Sessions	Loan of Headsets with Individual Use of IVR-N for Two More Weeks	Qualitative Interview
Patient 1	X	X	X
Spouse 1	X	X	X
Patient 2	X	X	X
Patient 3	X	X	X
Spouse 2	X	X	X
Patient 4	X		X
Patient 5	X	X	
Spouse 3	X	X	X
Patient 6	X		
Patient 7	X		X
Spouse 4	X		X

X indicates that the person participated in that part of the study. One patient died before the interview could be conducted, another patient declined interview.

**Table 2:** Clinical characteristics of the participating patients and spouses.

	Patients, n = 7	Spouses, n = 4
Sex, women/men, n	2/5	4/0
Age, mean value (min, max)	76 (68, 84)	76 (69, 78)
Living with spouse, n	6	4
Disease, n *		
Cancer	4	
Lung disorder	3	
Cardiovascular disease	2	
IPOS (mean score, SD)		
Physical symptoms, scale range 0–40	10.7 (4.7)	
Emotional concerns, scale range 0–16	6.0 (2.6)	
Communication/practical issues, scale range 0–12	1.6 (2.4)	
Total score	19.7 (5.5)	

IPOS: Integrated Palliative Care Outcome Scale, scale range 0–68. SD: standard deviation. * Two patients had two concurrent severe diseases.

**Table 3:** Description of the natural environments chosen by the patients and their spouses.

Environment	Number of Viewings	
	Patients, n = 7	Spouses, n = 4
Seagull with eggs	3	0
Birch pasture with cows	3	1
Winter campfire	3	1
Seaside in winter sun	2	0
Riverbank	1	0
Bay	1	2
Mire	1	0
Seaside cliffs	0	2
Pine forest in morning fog	0	1
Meadow on a summer night	0	1
Total viewings	14	8

**Table 4:** Overview of themes and subthemes.

Theme	Subtheme
Being in a place where tranquility becomes possible	Being in another world
	Finding peace and calmness
	Being released from emotional suffering
	Being released from bodily suffering
Connecting to nature via past experiences	Reliving past experiences
	Feeling at home
	Grieving lost opportunities
Being prevented from engaging fully with nature	Feeling a lack of ease in an overly calm environment
	Finding IVR-N to be a complement rather than a replacement

## Data Availability

The data supporting the findings of this study are available from the corresponding authors upon reasonable request. Access to the interview data is restricted due to ethical considerations and the need to protect the privacy and confidentiality of the participants.

## Supplementary Materials

The following supporting information can be downloaded at https://www.mdpi.com/article/10.3390/healthcare14213637/s1, File S1: COREQ (COnsolidated criteria for REporting Qualitative research) Checklist; File S2: Information for research participants/patients; File S3: Information for research participants/next of kin; File S4: Interview Guides; File S5: The patients’ individual and mean values of scoring symptoms before and after two separate IVR-N sessions; File S6: The spouses individual and mean values of scoring mental state before and after two separate IVR-N sessions; File S7: Meaning units, condensed meaning units, codes, sub-themes and themes from content analysis; File S8: Overview of patients and spouses’ representations in respective themes; File S9: Mixed-method display; File S10: Descriptions of the Authors with Reflexivity and Positionality Statements. Ref. [43] is cited in File S1.
