# Supplementary material for: Lived and Self-Reported Experiences of Immersive Virtual Nature Environments Among Patients and Spouses in Palliative Home Care: An Exploratory Mixed-Methods Feasibility Study
Source: Healthcare (Basel). 2026 Oct 27;14(21):3637. doi: 10.3390/healthcare14213637 (PMC13762592; doi:10.3390/healthcare14213637)
Supplement: Supplementary file 1 [file healthcare-14-03637-s001.zip › File S10. Author Reflexivity and Positionality Statements.pdf]

Supplementary File S10. Descriptions of the Authors with Reflexivity and Positionality Statements.

	**CN**	**LSJ**	**KL**
Positionality	The first author is a 65-year-old female registered nurse with extensive clinical and academic experience in geriatric and palliative care. Her professional background includes many years of work within geriatric care and palliative care, as well as a joint clinical-academic appointment at a hospice, between 2022 and 2024. She obtained her PhD in 2018 with a dissertation exploring spirituality and religiosity among the oldest old, employing both quantitative and qualitative research methods.<br><br>Since 1997, she has been involved in undergraduate nursing education, holding a particular responsibility for courses in palliative care. Throughout her academic career, she has developed a strong interest in the influence of care environments on health, well-being, and quality of care. This interest informed her engagement in the Interreg Aurora program NATUREACH, where she joined the work package focusing on the use of IVR-N in Palliative Care.	A 68-year old female physician with clinical specializing in internal medicine, cardiology, and occupational and environmental medicine. PhD in medicine 1993, since 2019 professor in Occupational medicine at Umeå University.<br>Senior consultant in occupational and environmental medicine since 2004. Now retired, but still active as senior professor part-time.<br><br>Twenty years of experience in research on the health effects of natural environments. Primarily a quantitative researcher but has also participated in some qualitative studies as co-author. The research has often been interdisciplinary in nature.<br><br>Co-applicant for the Interreg Aurora program NATUREACH and thereby representing Umeå University. In this role, responsible for the work package focusing on the use of IVR-N in Palliative Care.	A 66-year-old female enrolled nurse with extensive clinical and academic experience in geriatric care across institutional and home care settings. She obtained her PhD in 2009 and was appointed Associate Professor in 2022.<br><br>Her research primarily focuses on promoting health and quality of life through non-pharmacological interventions for older adults and individuals living with chronic health conditions. The research has predominantly involved intervention studies, but has also encompassed qualitative interview studies, health economic evaluations, and register-based research.<br><br>As a member of the broader NATUREACH research project, the author had prior knowledge of the intervention and its intended outcomes. The author has also been involved in evaluating another client subgroup using IVR-N within the NATUREACH project.
Clinical experience of palliative care	Yes	No	Yes

Previous experience of VR-technologies	No	No	No
Personal interest in Nature-Based Recreation	Yes	Yes	Yes

Reflexivity Statement

Catharina Norberg

The first author is a registered nurse and university lecturer with extensive experience in nursing education and research within the fields of geriatric and palliative care. This professional background provided valuable insights into the study context and facilitated an in-depth understanding of participants' experiences. At the same time, the authors were aware that their prior knowledge, experiences, and professional perspectives could influence data interpretation.

Throughout the research process, reflexivity was actively maintained through ongoing discussions among the authors, critical reflection on emerging interpretations, and collaborative data analysis. The research team comprised individuals with different professional and academic backgrounds, which contributed to challenging assumptions and enhancing analytical rigor. Efforts were made to remain open to participants' perspectives and to ensure that interpretations were grounded in the data rather than in pre-existing understandings.

To enhance the credibility and trustworthiness of the study, systematic data analysis, repeated engagement with the data, and ongoing discussions among the research team were conducted throughout the analytical process. Researcher's reflexivity, collaborative interpretation of the findings, and transparent reporting of methodological decisions further strengthened the study's rigor. Together, these strategies supported the development of findings that were closely grounded in participants' experiences and informed by a transparent and systematic analytical process.

Reflexivity Statement

Lisbeth Slunga Järvholm

Researcher Background and Epistemological Position

The co-author in this study is a 68-year-old female physician and Senior Professor of Occupational Medicine at Umeå University, Sweden. With a clinical background specializing in internal medicine, cardiology, and occupational and environmental medicine, her career spans several decades of bridging clinical practice with academic research. After earning a PhD in medicine in 1993, she has served as a senior consultant in occupational and environmental medicine since 2004, and as a professor in occupational medicine since 2019. Although officially retired from full-time duties, she remains actively engaged in research on a part-time basis.

Throughout her career, the researcher’s epistemological foundation has primarily been rooted in quantitative methodology, backed by twenty years of experience investigating the health effects of natural environments. However, her research approach has frequently been interdisciplinary, and she has previously co-authored qualitative studies. This blended experience provides a unique vantage point: a strong foundation in empirical, measurable data paired with a growing appreciation for the nuanced, experiential insights that qualitative inquiry offers.

Role in the Project and Potential Biases

In the context of the Interreg Aurora program "Natureach," the researcher serves as a co-applicant

representing Umeå University and leads the work package evaluating the use of Immersive Virtual Reality Nature (IVR-N) in palliative care.

Several factors regarding the researcher's background may have influenced the conceptualization, data collection, and analysis within this mixed-methods study:

- **Clinical Lens:** A background in cardiology and internal medicine naturally instills a focus on physiological outcomes, symptom management, and patient safety. In a qualitative or mixed-methods evaluation of palliative care, this could predispose the researcher to look for therapeutic or clinical efficacy, potentially overshadowing broader psychological, existential, or purely subjective user experiences.
- **Preconceptions of Nature's Health Benefits:** With two decades of dedicated research into how natural environments impact human health, the researcher holds a strong, evidence-based belief in the positive therapeutic potential of nature. This baseline assumption required conscious mitigation during data analysis to ensure that any negative, neutral, or distressing patient reactions to the IVR-N experience were captured and analyzed with equal weight and rigor.
- **Quantitative Baseline vs. Qualitative Openness:** Being predominantly trained as a quantitative scientist, there is an inherent tendency to seek patterns, standardization, and generalizability. To maintain qualitative integrity, the researcher actively collaborated with co-authors to ensure that the qualitative components prioritized the depth, unique context, and lived experiences of the participants, rather than reducing them to rigid, quantifiable variables.

Mitigation and Trustworthiness

To ensure the trustworthiness, credibility, and confirmability of the qualitative findings, several reflexive strategies were employed. The researcher engaged in collaborative data analysis with co-investigators who possessed extensive qualitative expertise. This interdisciplinary dialogue served as an analytical check, challenging potential quantitative or purely clinical biases, and ensuring that the thematic analysis remained deeply grounded in the participants' actual accounts. By maintaining explicit awareness of her professional trajectory and pre-existing views on environmental therapeutics, the researcher sought to transform these potential biases into a tool for nuanced, critical insight.

Reflexivity Statement

Kristina Lämås

The author is a 66-year-old female enrolled nurse and Associate Professor with extensive clinical and academic experience in geriatric care across institutional and home care settings. She obtained her PhD in 2009 and was appointed Associate Professor in 2022. Her professional background encompasses many years of experience working with older adults, contributing to a strong understanding of the challenges and opportunities associated with ageing, chronic illness, and care provision in later life including palliative care.

Her research primarily focuses on promoting health, well-being, and quality of life through non-pharmacological interventions among older adults and individuals living with chronic health conditions. Her research experience includes intervention studies, qualitative interview studies, health economic evaluations, and register-based research. Across these methodological approaches, she has developed particular expertise in exploring participants' experiences, perceptions, and meaning-making processes in relation to health-promoting interventions.

Within the present mixed methods study, the author was primarily involved in the qualitative components of the research, including the interpretation and analysis of participants’ experiences. Her clinical and

research background contributed to a contextual understanding of the population under study and informed the interpretation of the qualitative findings. This background may have facilitated sensitivity to nuances in participants' narratives; however, it also carried the potential risk of pre-understanding influencing data interpretation.

As a member of the broader NATUREACH research project, the author had prior knowledge of the intervention under investigation and its intended outcomes. Furthermore, she had previously participated in the evaluation of another client subgroup using IVR-N within the NATUREACH project. While this involvement provided valuable contextual insight into the intervention and study setting, it also positioned the author as an insider within the research programme. To address potential bias, reflexive awareness was maintained throughout the research process, including during data analysis and interpretation. Interpretations were continuously discussed within the multidisciplinary research team, allowing different perspectives to challenge assumptions and strengthen analytical rigour.

From a constructivist perspective, the author acknowledges that knowledge is co-produced through interactions among researchers, participants, and the research context. Consequently, her professional experiences, disciplinary background, and familiarity with the intervention inevitably shaped how participants' accounts were understood and interpreted. Throughout the study, efforts were made to critically reflect upon these influences, to remain open to unexpected findings, and to ensure that interpretations were grounded in the participants' narratives rather than in prior expectations regarding the intervention.
