# Supplementary material for: Lived and Self-Reported Experiences of Immersive Virtual Nature Environments Among Patients and Spouses in Palliative Home Care: An Exploratory Mixed-Methods Feasibility Study
Source: Healthcare (Basel). 2026 Oct 27;14(21):3637. doi: 10.3390/healthcare14213637 (PMC13762592; doi:10.3390/healthcare14213637)
Supplement: Supplementary file 1 [file healthcare-14-03637-s001.zip › File S2. Information for participants-patients.pdf]

Information for research participants/patients

We invite you to participate in a research project. This document contains information about the project and what participation entails.

What is the project about, and why are you being asked to participate?

The "Virtual nature environments" project aims to make nature experiences possible even when you are ill and need to spend most of your time indoors. Virtual reality (VR) is a technology that enables virtual experiences and creates a sense of presence in the natural environment displayed via VR headsets. Previous studies have shown that VR can complement other treatment methods in palliative care. The project's overall aim is to investigate the value and benefits of virtual nature environments in palliative care.

You are being asked to participate in the study because you are admitted as patient to a palliative care unit. The unit manager at the care unit has approved the study's implementation at the facility. We received your contact details from the healthcare staff.

The research project is conducted by Umeå University, which is the organization responsible for the project. The research project has been approved by the Swedish Ethical Review Authority (reference number 2024-00433-01).

How is the project conducted?

Participating in the project involves taking part of and experiencing specially filmed nature environments by using VR headsets. There are several nature environments to choose from; each experience lasts approximately 15 minutes. You will experience virtual nature experience on three separate occasions with the assistance of a nurse. First, you will answer a questionnaire about your health. You will then be asked questions about your symptoms before and after the nature experience.

After these three occasions, you will be offered the opportunity to borrow the equipment for an additional two weeks. Upon completion of the project, you will be asked to participate in an individual interview regarding your experiences with the VR nature sessions. The interview – conducted as a conversation – is expected to take approximately 30 minutes and will be audio-recorded.

Potential consequences and risks of participating in the project

Participating in the project may contribute to increased well-being and help evoke positive memories of past nature experiences. Participation could also potentially cause some discomfort due to the equipment or memories triggered by the nature environments. The nurses and researchers you meet are experienced in discussing sensitive topics and will be present during the first three VR nature sessions. You will also have the opportunity to share

your experiences with them of the virtual nature environments. If problems arise or if you experience discomfort, you may withdraw from the study at any time.

What happens to your data?

The project will collect and record information about you, but all data will be assigned a code number, meaning the collected information cannot be linked to your identity. Personal data, contact details, and consent forms will be stored separately and securely under lock and key. Audio files and interview transcripts will be assigned code numbers and stored on a password-protected computer. Once the study is concluded, all data will be archived on a secure, locked storage device or a secure server. Personal data will be deleted when no longer needed for the project.

Only the researchers responsible for the project will have access to the collected material. All data is handled in a manner that prevents unauthorized access. Information you provide regarding the nature experience and during the subsequent interview will be treated confidentially; this means it cannot be linked to you personally when the research results are presented.

The data controller, Umeå University, is responsible for your personal data. Under the EU General Data Protection Regulation (GDPR), you have the right to access the data concerning you that is being processed in the project free of charge and, if necessary, to have any errors corrected. You may also request that your data be deleted or that the processing of your personal data be restricted. However, the right to erasure and restriction of processing does not apply when the data is necessary for the research in question.

If you wish to access the data, please contact the project's principal investigator, Lisbeth Slunga Järvholm at Umeå University. The Data Protection Officer can be reached at 090-786 50 00. If you are dissatisfied with how your personal data is processed, you have the right to lodge a complaint with the Swedish Authority for Privacy Protection.

How will you receive information about the project results?

Once your participation in the project has concluded, the responsible nurse will provide you with a summary of your results. If you wish to receive detailed information about your results or the results of the study as a whole, please contact the principal investigator, Lisbeth Slunga Järvholm. Any incidental findings unrelated to the project's purpose will not be used in the data analysis but will remain part of the collected data; this information will be stored securely in a locked area or on a secure server, separate from your personal data.

Insurance and compensation

Patient insurance coverage applies during your participation in the study. No compensation is provided for participating in the project.

Participation is voluntary

Your participation is voluntary, and you may choose to withdraw at any time. If you choose not to participate or wish to withdraw, you do not need to provide a reason, and it will not affect your future care or treatment.

If you wish to withdraw from the project, please contact the person responsible for the project (see below).

Project leadership

The project leader is Lisbeth Slunga Järvholm; see contact details below.

Lisbeth Slunga Järvholm
Professor, Senior Consultant
Department of Public Health and Clinical Medicine
Umeå University
070–5685258
lisbeth.slunga-jarvholm@umu.se
