# Supplementary material for: Lived and Self-Reported Experiences of Immersive Virtual Nature Environments Among Patients and Spouses in Palliative Home Care: An Exploratory Mixed-Methods Feasibility Study
Source: Healthcare (Basel). 2026 Oct 27;14(21):3637. doi: 10.3390/healthcare14213637 (PMC13762592; doi:10.3390/healthcare14213637)
Supplement: Supplementary file 1 [file healthcare-14-03637-s001.zip › File S4. Interview guide.pdf]

Interview Guides

Semi-Structured Individual Interviews with Patients and Family Members

Interview approach: We aim to conduct individual interviews with patients and family members. However, we remain flexible and open to arrangements that best suit the participants and their specific circumstances. Semi-structured interviews will be used to encourage participants to share their experiences in a narrative manner. When needed, probing questions will be used, such as: *"Could you tell me more about that?"*, *"Could you clarify what you mean?"*, or *"Have I understood correctly that you are saying…?"*

At the end of each interview, participants will be asked: *"Is there anything else you would like to tell us about your experience of using the VR equipment and viewing the recorded natural environments?"*

Interviews will be conducted in the participant's home, at the palliative care unit, or at another location chosen by the participant. All interviews will be audio-recorded using dedicated recording equipment and subsequently transferred to a password-protected computer belonging to CN. The audio recordings will then be deleted from the recording device. Interviews will be transcribed verbatim and de-identified. All data, including interview transcripts, will be coded. The interviews will focus on participants' experiences and perceptions of IVR-N

Interview Guide - Patients

- Can you tell us about your previous experiences of being in nature?
- Can you describe your experience of viewing nature environments through VR?
- Is there anything else you would like to say about your experience?
- Did any particular thoughts arise while you were using VR?
- How did you experience your body during the VR session?
- Did VR influence your experience of symptoms or discomfort? If yes, could you describe, in what way?
- How did you experience the different environments presented in VR?
- Were any of the environments particularly meaningful or influential for you?
- Are there other environments you would have liked to experience in addition to those available in the VR headset?

- What were you thinking or feeling while using VR? (e.g., frustration, sadness, joy)
- Did you experience any discomfort during or after the VR session? If yes, could you describe what kind of discomfort and in what way it affected you? (This may include discomfort related to the equipment, such as weight, neck strain, discomfort while sitting or lying down, difficulties wearing glasses, etc.)
- Has the use of VR been helpful to you in coping with your current situation?
- Would you like to continue using this VR equipment?

Interview Guide - Family Members

- Can you tell us about your previous experiences of being in nature?
- Can you describe your experience of viewing nature environments through VR?
- Is there anything else you would like to say about your experience?
- Did any particular thoughts arise while you were using VR?
- How did you experience your body during the VR session?
- How did you experience the different environments presented in VR?
- Were any of the environments particularly meaningful or influential for you?
- Are there other environments you would have liked to experience in addition to those available in the VR headset?
- What were you thinking or feeling while using VR? (e.g., frustration, sadness, joy)
- Did you experience any discomfort during or after the VR session? If yes, could you describe what kind of discomfort and how it affected you? (This may include discomfort related to equipment, such as weight, neck strain, discomfort while sitting or lying down, difficulties wearing glasses, etc.)
- Has the use of VR been helpful to you in coping with your current situation?
- Would you like to continue using this VR equipment?
- Have you made any particular observations regarding your relative's (e.g., friend, partner, parent) use of the VR headset?
