# Supplementary material for: Lived and Self-Reported Experiences of Immersive Virtual Nature Environments Among Patients and Spouses in Palliative Home Care: An Exploratory Mixed-Methods Feasibility Study
Source: Healthcare (Basel). 2026 Oct 27;14(21):3637. doi: 10.3390/healthcare14213637 (PMC13762592; doi:10.3390/healthcare14213637)
Supplement: Supplementary file 1 [file healthcare-14-03637-s001.zip › File S5. Patient Data.pdf]

Supplementary File S5. The patients' individual and mean values of scoring symptoms before and after two separate IVR-N sessions.

Patients	NRS (0-10)															
	Pain				Anxiety				Nausea				Breathless			
	Pre 1	Post 1	Pre 2	Post 2	Pre 1	Post 1	Pre 2	Post 2	Pre 1	Post 1	Pre 2	Post 2	Pre 1	Post 1	Pre 2	Post 2
Patient 1	0	0	1	0	1	2	2	1	0	0	0	0	1	0	2	0
Patient 2	0	0	0	0	3	2	2	1	0	0	3	2	5	2	2	1
Patient 3	0	0	0	0	0	0	0	0	0	0	0	0	4	4	4	4
Patient 4	0	0	1	1	0	0	3	3	0	0	0	0	5.5	5.5	3	3
Patient 5	2	2	3	2	2	2	2	2	2	0	0	0	0	2	2	2
Patient 6	0	0	0	0	7	2	5	2	0	0	0	0	4	0	5	0
Patient 7	5	5	4	4	0	0	0	0	0	0	0	0	0	0	1	1
Mean value (SD)	1.0 (1.9)	1.0 (1.9)	1.3 (1.6)	1.0 (1.5)	1.9 (2.5)	1.1 (1.1)	2.0 (1.1)	1.3 (1.1)	0.3 (0.8)	0 (0)	0.4 (1.1)	0.3 (0.8)	2.8 (2.4)	1.9 (2.2)	2.7 (1.4)	1.6 (1.5)

NRS = Numeric Rating Scale, a high value indicates a high degree of symptom.
