# Supplementary material for: Lived and Self-Reported Experiences of Immersive Virtual Nature Environments Among Patients and Spouses in Palliative Home Care: An Exploratory Mixed-Methods Feasibility Study
Source: Healthcare (Basel). 2026 Oct 27;14(21):3637. doi: 10.3390/healthcare14213637 (PMC13762592; doi:10.3390/healthcare14213637)
Supplement: Supplementary file 1 [file healthcare-14-03637-s001.zip › File S6. Spouse Data.pdf]

Supplementary File S6. The spouses individual and mean values of scoring mental state before and after two separate IVR-N sessions.

Mental state scoring (1-10)		Spouses				Mean value (SD)
		Spouse 1	Spouse 2	Spouse 3	Spouse 4	
Tense/relaxed	Pre 1	3	8	10	8	7.2 (3.0)
	Post 1	8	9	10	8	8.8 (1.0)
	Pre 2	8	8	8	5	7.2 (1.5)
	Post 2	9	10	9	4	8.0 (2.7)
Exhausted/alert	Pre 1	4	5	9	4	5.5 (2.4)
	Post 1	8	5	10	2	6.2 (3.5)
	Pre 2	9	5	6	2	5.5 (2.9)
	Post 2	9	4	9	1	5.8 (3.9)
Sad/happy	Pre 1	5	6	9	6	6.5 (1.7)
	Post 1	9	9	10	5	8.2 (2.2)
	Pre 2	9	7	6	3	6.2 (2.5)
	Post 2	9	4	9	3	6.2 (3.2)
Irritated/harmonious	Pre 1	5	9	9	7	7.5 (1.9)
	Post 1	8	9	10	8	8.8 (1.0)
	Pre 2	8	2	9	3	5.5 (3.5)
	Post 2	8	9	9	6	8.0 (1.4)
Restless/peaceful	Pre 1	4	9	9	4	6.5 (2.9)
	Post 1	9	10	10	7	9.0 (1.4)
	Pre 2	9	9	6	2	6.5 (3.3)
	Post 2	10	9	10	6	8.8 (1.9)
Distracted/clear-headed	Pre 1	5	8	10	5	7.0 (2.4)
	Post 1	8	9	10	9	9.0 (0.8)
	Pre 2	8	9	6	1	6.0 (3.6)
	Post 2	9	9	10	6	8.5 (1.7)

The mental state scale is a 10-point scale ranging from 1 to 10, a high score indicates a favorable mental state. Spouse 1 is related to Patient 1, Spouse 2 to Patient 3, Spouse 3 to Patient 5, and Spouse 4 to Patient 7.
