# Supplementary material for: Lived and Self-Reported Experiences of Immersive Virtual Nature Environments Among Patients and Spouses in Palliative Home Care: An Exploratory Mixed-Methods Feasibility Study
Source: Healthcare (Basel). 2026 Oct 27;14(21):3637. doi: 10.3390/healthcare14213637 (PMC13762592; doi:10.3390/healthcare14213637)
Supplement: Supplementary file 1 [file healthcare-14-03637-s001.zip › File S7. Qualitative content analysis.pdf]

Begränsad delning

Supplementary File S7. Meaning units, condensed meaning units, codes, sub-themes and themes from content analysis

**Meaning unit**	**Condensed meaning unit. Description close to the text**	**Sub-theme**	**Theme**
"…having a break and thinking about something else is very important for me. It's easy to get stuck in these rather dark thoughts. That's quite hard. But that feeling disappeared after a little while when I was sitting there with those glasses on."	Distraction from troubling thoughts, including concerns about death and the end of life.	Being released from emotional suffering	Being in a place where tranquility becomes possible
"We could relax, we could sit there for about fifteen minutes… just look at those images and forget all the boring stuff that was behind us… we got a break, that's what I felt." (Spouse 3)	To shift attention to something else and leave distressing thoughts behind.		
… these last few days I've been experiencing terrible shortness of breath. It feels like I'm going to suffocate, like there's no way to get air in or out. And then, being able to disconnect from that just by sitting and looking out over the sea, or something like that… that amazed me.	It was surprising that nature experience through VR-glasses made it possible to unwind feelings of breathlessness	Being released from bodily suffering	
I felt relaxed and almost unaware of my body…it was soothing and calming to look at the images —especially when there was activity in the environment.	I felt completely relaxed and forgot my body, the experience was peaceful and soothing.		
