# Supplementary material for: Lived and Self-Reported Experiences of Immersive Virtual Nature Environments Among Patients and Spouses in Palliative Home Care: An Exploratory Mixed-Methods Feasibility Study
Source: Healthcare (Basel). 2026 Oct 27;14(21):3637. doi: 10.3390/healthcare14213637 (PMC13762592; doi:10.3390/healthcare14213637)
Supplement: Supplementary file 1 [file healthcare-14-03637-s001.zip › File S8. Participants representation in each theme.pdf]

Supplement File S8. Overview of patients and spouses' representations in respective themes.

Theme	Patient-representation in theme	Spouse-representation in theme
Being in a place where tranquility becomes possible	Patient: 1, 2, 3, 4, 7	Spouse: 1, 2, 3, 4
Connecting to nature via past experiences	Patient: 1, 2, 3, 4, 7	Spouse: 1, 2, 3, 4
Being prevented from engaging fully with nature	Patient: 1, 4, 7	Spouse: 1, 2, 4
