# Supplementary material for: Lived and Self-Reported Experiences of Immersive Virtual Nature Environments Among Patients and Spouses in Palliative Home Care: An Exploratory Mixed-Methods Feasibility Study
Source: Healthcare (Basel). 2026 Oct 27;14(21):3637. doi: 10.3390/healthcare14213637 (PMC13762592; doi:10.3390/healthcare14213637)
Supplement: Supplementary file 1 [file healthcare-14-03637-s001.zip › File S9. Mixed method display.pdf]

Suppl. File S9. Mixed-method display

The table show the links between quantitative finding and qualitative findings. The links represent integration at group level.

Study group	Descriptive data, mean change	Subtheme
**Patients (NRS) (n=7)**		
• Pain<br>○ First session<br>○ Second session	 0.0<br>-0.1	Being released from bodily suffering
• Anxiety<br>○ First session<br>○ Second session	 -1.4<br>-0.7	Finding peace and calmness
• Nausea<br>○ First session<br>○ Second session	 -0.3<br>-0.1	Being released from bodily suffering
• Breathless<br>○ First session<br>○ Second session	 -0.9<br>-1.1	Being released from bodily suffering
**Spouses (Mental state) (n=4)**		
Tense/relaxed<br>○ First session<br>○ Second session	 1.0<br>0.8	Finding peace and calmness
Exhausted/alert<br>○ First session<br>○ Second session	 0.7<br>0.3	Finding peace and calmness
Sad/happy<br>○ First session<br>○ Second session	 1.7<br>0.0	Being released from emotional suffering
Irritated/harmonious<br>○ First session<br>○ Second session	 1.3<br>2.5	Being released from emotional suffering
Restless/peaceful<br>○ First session<br>○ Second session	 2.5<br>2.3	Finding peace and calmness
Distracted/clearminded<br>○ First session<br>○ Second session	 2.0<br>2.5	------------

NRS = Numeric Rating Scale, ranging from 0 to 10, a negative value of the mean change indicates decreased symptoms.

The Mental State scale, ranging from 1 to 10, a positive value of the mean change indicates a more favourable mental state.
